# It’s all in the timing: delayed feedback in autism may weaken predictive mechanisms during contour integration

**DOI:** 10.1152/jn.00058.2024

**Published:** 2024-07-03

**Authors:** Emily J. Knight, Ted S. Altschuler, Sophie Molholm, Jeremy W. Murphy, Edward G. Freedman, John J. Foxe

**Affiliations:** ^1^The Frederick J. and Marion A. Schindler Cognitive Neurophysiology Laboratory, Department of Neuroscience, The Del Monte Institute for Neuroscience, University of Rochester School of Medicine and Dentistry, Rochester, New York, United States; ^2^Development and Behavioral Pediatrics, Golisano Children's Hospital, University of Rochester School of Medicine and Dentistry, Rochester, New York, United States; ^3^The Cognitive Neurophysiology Laboratory, Department of Pediatrics and Neuroscience, Albert Einstein College of Medicine, Bronx, New York, United States; ^4^Program in Cognitive Neuroscience, Departments of Psychology & Biology, City College of the City University of New York, New York, United States; ^5^Department of Neuroscience, Brown University, Providence, Rhode Island, United States

**Keywords:** autism spectrum disorder, illusory contours, object recognition, visual-evoked potentials, visual feedback

## Abstract

Humans rely on predictive and integrative mechanisms during visual processing to efficiently resolve incomplete or ambiguous sensory signals. Although initial low-level sensory data are conveyed by feedforward connections, feedback connections are believed to shape sensory processing through automatic conveyance of statistical probabilities based on prior exposure to stimulus configurations. Individuals with autism spectrum disorder (ASD) show biases in stimulus processing toward parts rather than wholes, suggesting their sensory processing may be less shaped by statistical predictions acquired through prior exposure to global stimulus properties. Investigations of illusory contour (IC) processing in neurotypical (NT) adults have established a well-tested marker of contour integration characterized by a robust modulation of the visually evoked potential (VEP)—the IC-effect—that occurs over lateral occipital scalp during the timeframe of the visual N1 component. Converging evidence strongly supports the notion that this IC-effect indexes a signal with significant feedback contributions. Using high-density VEPs, we compared the IC-effect in 6- to 17-yr-old children with ASD (*n* = 32) or NT development (*n* = 53). Both groups of children generated an IC-effect that was equivalent in amplitude. However, the IC-effect notably onset 21 ms later in ASD, even though initial VEP afference was identical across groups. This suggests that feedforward information predominated during perceptual processing for 15% longer in ASD compared with NT children. This delay in the feedback-dependent IC-effect, in the context of known developmental differences between feedforward and feedback fibers, suggests a potential pathophysiological mechanism of visual processing in ASD, whereby ongoing stimulus processing is less shaped by visual feedback.

**NEW & NOTEWORTHY** Children with autism often present with an atypical visual perceptual style that emphasizes parts or details over the whole. Using electroencephalography (EEG), this study identifies delays in the visual feedback from higher-order sensory brain areas to primary sensory regions. Because this type of visual feedback is thought to carry information about prior sensory experiences, individuals with autism may have difficulty efficiently using prior experience or putting together parts into a whole to help make sense of incoming new visual information. This provides empirical neural evidence to support theories of disrupted sensory perception mechanisms in autism.

## Graphical abstract

**Figure:**
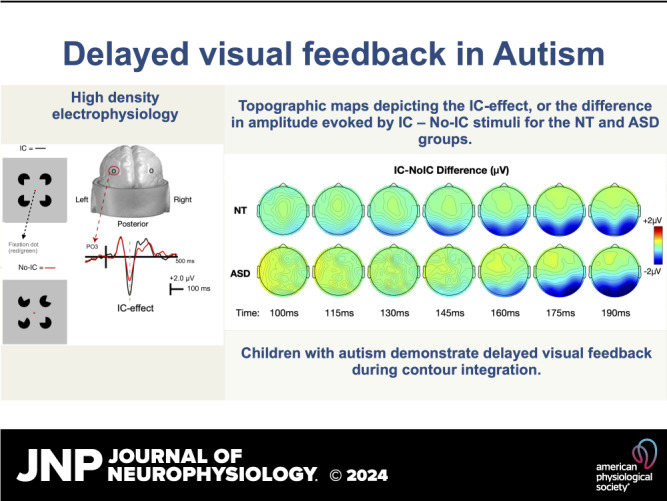

## INTRODUCTION

Individuals with autism spectrum disorder (ASD) are notable for an atypical cognitive style, often emphasizing parts rather than wholes (1). Enhanced perceptual processing of features (2), weakness in global processing (3), or weakened application of prior knowledge to the processing of incoming sensory data (4) have been offered as explanations of this characteristic imbalance. Here, we report on delayed feedback in the context of unaltered feedforward contributions to early perceptual processing, providing neurophysiological evidence in support of these theories.

Major challenges encountered by the brain in the creation of visual representations of objects from the natural world include: *1*) missing information—the retinal surface is interrupted by the optic nerve and by a network of vasculature (5); *2*) ambiguity—one object viewed from different angles projects different shapes upon the retina (6); *3*) poor conditions—environmental conditions are seldom optimal, such that objects are often seen under poor lighting conditions or are partially occluded by other objects. Both the poor conditions and ambiguity of incoming signals are thought to be resolved via interactions between sensory representations and the extensive prior exposure to a sensory-rich environment that has shaped the development of the visual system (7).

Although the visual system is characterized as a hierarchy, with lower cortex encoding the most basic features, inputting to successively higher areas that encode ever more complex combinations (8), information moves rapidly both up and down the system (9). Feedforward pathways play a key role in extracting and integrating sensory data (10), whereas feedback projections are thought to convey statistical predictions based upon likelihoods of particular stimulus configurations (11). This feedback, in turn, shapes the feedforward information via an automatic and rapid iterative process that disambiguates the representation of incoming data (12–16).

Feedforward and feedback projections in the visual cortex of nonhuman primates originate and terminate in different layers of cortex (9) and crucially, they reach their mature targets over considerably different developmental time courses (17). Prolonged maturation of feedback projections is also seen in humans (18), establishing a neural basis for the selective vulnerability of the connections they make and their role in visual processing, necessitating exploration of the role of feedback in clinical populations manifesting atypical development.

Toward that end, visual binding paradigms offer an accessible vehicle to probe the integrity of these projections in various populations. Binding of elements in the formation of visual object representations has been associated with feedback in nonhuman primates (19). In humans, delays specific to feedback connections have been associated with visual binding deficits in schizophrenia independently of altered timing in feedforward connections (20). Contour integration, involving the filling-in between fragments of contours, is one such binding task (21, 22), and this mechanism has been extensively studied using Kanizsa illusory contours (IC) (23). A modulation of the visual-evoked potential (VEP) indexes this process in neurotypical (NT) adults and children (21, 24, 25). This modulation onsets within ∼90 ms of stimulus presentation and peaks at around 150 ms in neurotypical adults. This modulation has been termed the IC-effect, and is associated with automatic filling-in of object boundaries (26).

This IC-effect has been localized to the lateral occipital complex (LOC) (21, 27–29), a group of extrastriate regions that encodes information about coherent objects, independent of the features of which they are comprised (30). Converging evidence from animal and human work strongly supports a feedback-driven model of contour integration. Studies in nonhuman primates and mice that have indexed the precise timing of contour integration imply a significant role of feedback connections in this type of processing (12, 31). For example, Lamme and coworkers (12, 32) demonstrated that figure-ground segregation processing is evident in V1 neurons of nonhuman primates at ∼80–100 ms following stimulus delivery (a significant delay relative to initial afferent inputs to V1), analogous to human illusory-contour effects (21, 33). In a follow-up study, using a backward masking paradigm in awake behaving macaques accompanied by neural recordings from V1 neurons, they found that masking disrupts figure-ground segregation via selective interruption of the recurrent interactions between V1 and higher-order extrastriate regions (34). Likewise, in humans, substantial work has shown that VEP modulations due to contour integration are detectable within 40–50 ms of initial afference in V1. Initial afference to V1 is thought to be dominated by representation of local features processed in lower visual areas, feeding forward through the system (35). Rapidly thereafter, higher-order integrative cortices receive initial afferent inputs and begin to convey information about the global scene back to lower levels (15, 16, 36–39). As in nonhuman primates, these contour integration-related VEP modulations are largely driven by feedback inputs from higher-order LOC ventral visual stream areas (21, 24, 26, 33, 40, 41). Additional compelling evidence for the role of feedback in illusory contour integration comes from direct manipulation of the circuitry in humans using transcranial magnetic stimulation (TMS) to disrupt signaling in V1/V2 and the LOC while participants were tasked with discriminating Kanizsa illusory contour stimuli. In this study (39), Wokke et al. elegantly demonstrated that TMS disruption of LOC activity between 100 and 122 ms significantly reduced illusory contour discrimination. In contrast, TMS to hierarchically early visual cortex (V1 and V2) was only disruptive to illusory contour discrimination only at 160–162 ms, after these areas began to receive feedback from LOC. Thus, it can be concluded that illusory contour modulations reflect an iterative (or resonant) feedback process between LOC and early visual cortex (e.g., V1) (26).

Here, we set out to make use of the exquisitely time-sensitive metric of electrophysiologic contour integration to investigate these feedback-dominated binding processes across a range of stimulus sizes in a cohort of 6- to 17-yr-old participants with ASD. Our central thesis was that these feedback processes would be delayed or disrupted in autism spectrum disorder, offering a potential neural mechanism for the proposed perceptual integration deficits associated with this condition.

## MATERIALS AND METHODS

### Participants

Fifty-seven NT and 38 ASD individuals aged 6 to 17 yr participated. Their sex, age, nonverbal IQ scores, and other pertinent descriptive data are provided in Table 1. Average age and nonverbal IQ scores did not differ between groups. There was a male predominance in the ASD group relative to the control group. Data from four neurotypical and six ASD participants were excluded either due to poor data quality as evidenced by rejection of greater than 50% of trials or for neuropsychological diagnoses uncovered following recording (in the NT group), resulting in a final cohort of 53 NT and 32 ASD participants. Participants provided informed assent and their parent or guardian gave informed consent. The City College of the City University of New York and Albert Einstein College of Medicine Institutional Review Boards approved all procedures.

**Table 1.** Participant characteristics

	ASD (*n* = 32)	NT (*n* = 53)	Significance (*P*)
Age	11.7 ± 2.3	11.7 ± 2.9	*P* = 0.995
Non-verbal IQ^a^	104.5 ± 18.5	108.7 ± 12.2	*P* = 0.279
Sex	84% Male	50% Male	*P* < 0.001

Means ± standard deviation is presented for age and IQ. Percentage male is presented for sex.

a IQ data not available for *n* = 2 autism spectrum disorder (ASD) and *n* = 3 neurotypical (NT) subjects. Equal variances not assumed.

Exclusionary criteria for both groups included history of seizures, head trauma, intellectual disability (full-scale IQ < 70), schizophrenia, bipolar disorder or psychosis, and history of neurological disorder or an identified syndromic cause for ASD. Additional exclusionary criteria for neurotypical participants included diagnosis of attention-deficit hyperactivity disorder (ADHD), learning disability, other developmental disorder, or history of a developmental disorder in a first-degree relative. Participants were screened for normal or corrected-to-normal vision, hearing, and color vision. Diagnoses of ASD were made on the basis of the Autism Diagnostic Observation Schedule (42) and Autism Diagnostic Interview-R (43) using DSM-IV criteria (assessments collected prior to the 2013 update to the DSM-V). Parents were asked to refrain from giving stimulant medication to their children in the 24 h preceding participation. Six remaining participants were treated with an antipsychotic and anxiolytic to treat anxiety (1), a mood stabilizer and antihypertensive (1) and a norepinephrine reuptake inhibitor (2) to treat ADHD, and a serotonin selective reuptake inhibitor (2) to treat anxiety.

### Stimulus and Task

**Figure 1.**
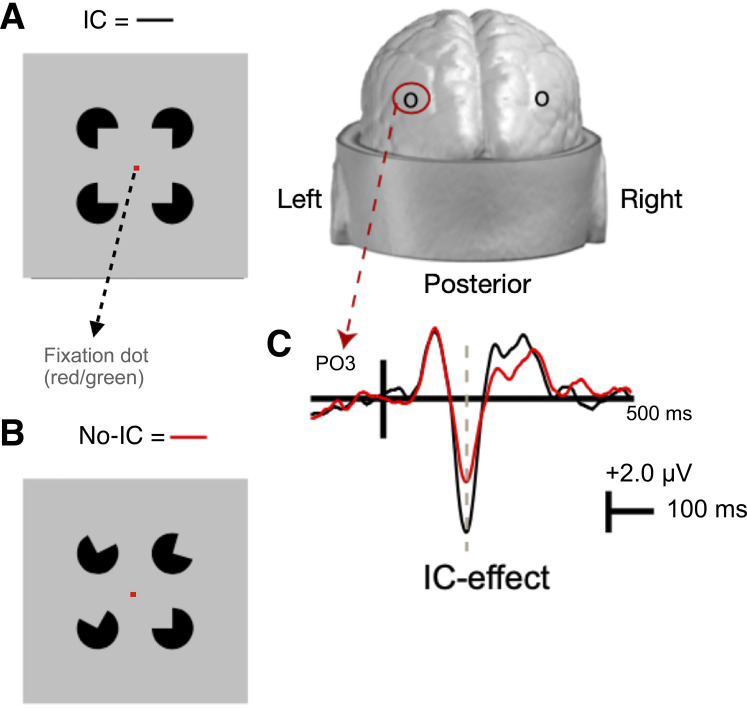
Representation of the illusory contour (IC; black) (*A*) and noncontour (No-IC; red) (*B*) stimuli as well as schematic of the a priori-defined electrodes of interest (PO3 and PO4) and sample visual-evoked potential tracings for IC and No-IC stimuli with the IC-effect marked by a dashed line (*C*).

Participants viewed a version of the Kanizsa illusion, consisting of four black “pacman-like” disks against a gray background (23) (Fig. 1). Each disk occupied one of four corners of a square-shaped array. Each had a 90° angle cut out of them—their “mouth.” When the mouths were angled such that their contours were collinear, the gap between the mouths appeared to fill-in, inducing the perception of a square (IC condition). When the mouths were not aligned, no illusion was induced (No-IC condition). In the No-IC condition, three of four inducers were rotated away from the center, and the mouth of the fourth inducer was aligned with one corner of the illusory square. The retinal eccentricity of the inducers was manipulated to produce three different size conditions for the illusory square 4°, 7°, and 10° of visual angle (extent) and the presentation of the three size conditions was varied randomly. Inducers for the three extents were 2.1°, 3.8°, and 5.6° diameter respectively, holding support ratio (the proportion of actual to perceived contour extent) constant.

**Figure 2.**
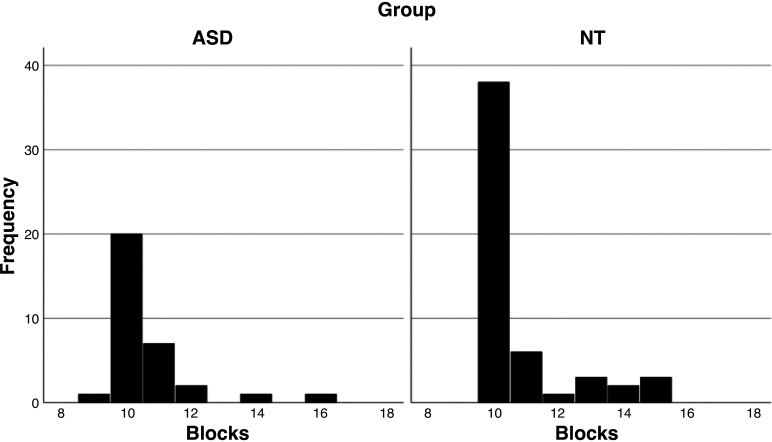
Histogram depicting the distribution of number of blocks completed for participants in the autism spectrum disorder (ASD, *left*) and neurotypical (NT, *right*) groups.

Participants sat in a dimly lit, sound-attenuated electrically shielded double-walled booth (Industrial Acoustics Company, Bronx, NY), 60 cm from a Dell Ultrasharp 1704FTP monitor with 1,280 × 1,024-pixel resolution or 75 cm from a ViewSonic VP2655 monitor with 1,680 × 1,050 pixel resolution (refresh rates 60 Hz). Stimulus durations were 500 ms with a randomly and equiprobably varied onset asynchrony between 800 and 1,400 ms. Ten to fifteen 3-min blocks were presented, with breaks as needed, until sufficient (>100 trials per condition) had been collected. Sufficient data collection was determined according to a computer-generated output of the number of trials presented during each block as well as the performance on the color change task. Continuous electrophysiological data collection was monitored in real time at the channel level to ensure maintenance of adequate tracings; however, trial-by-trial electrophysiological data were not evaluated in real-time. A histogram showing the distribution of block numbers is presented in Fig. 2. This distribution did not substantively differ between groups.

Explicit attention to ICs is not required to elicit electrophysiological indices of IC processing in NT adults (21) or children (44) when stimuli are centrally presented (i.e., foveated). Task instructions made no mention of the specific stimuli or the illusion. Instead, participants attended to a color-detection task involving the central fixation dot. Every 1–10 s, the dot changed from red to green for 160 ms on a random time-course uncorrelated with IC presentation. As the colors were chosen for an isoluminant plane of the DKL color-space (45), the change was imperceptible without foveating. Participants were asked to click a mouse button for each color-change. Six- to nine-year-old participants were directly observed to ensure fixation. Although only young children were directly observed by the experimenter in the booth, behavioral data for the color change task (which requires maintenance of foveation for accurate performance) was collected for all participants. Correct responses were reported out by the computer at the end of the block that provided a readout for the experimenter if children were not fixating sufficiently to accurately engage in the task. All children were reminded to maintain fixation if they were observed to be having difficulty. Both groups exhibited adequate performance on the color change task, performing well above chance, though participants with ASD performed less well (mean accuracy: ASD 84.2 ± 12.7%, NT 90 ± 12.5%, *t*_(79*)_ = 2.334; *P* = 0.022, Cohen’s *d* = 0.531, *four NT participants for whom behavioral data were not stored were excluded from this analysis).

Following administration of the main VEP experiment, a debriefing questionnaire assessed IC perception. When shown square-inducing stimuli like that used in the experiment, 100% of included participants identified the IC-condition as the “square.”

### Data Acquisition and Processing

Continuous electroencephalography (EEG) was recorded via a Biosemi ActiveTwo system from a 70-electrode montage, digitized at 512 Hz and referenced to the Common Mode Sense (CMS) and Driven Right Leg (DRL). EEG data were processed and analyzed offline using custom scripts that included functions from the EEGLAB (46) and ERPLAB Toolboxes (47) for MATLAB (the MathWorks, Natick, MA). Data were band-pass filtered using an IIR Butterworth filter between 0.1 and 50 Hz implemented in ERPLAB. Bad channels were manually and automatically detected and interpolated using EEGLAB spherical interpolation. Data were re-referenced to a frontal electrode (Fpz in the 10–20 system convention) and then divided into epochs starting 100 ms before the presentation of each IC/No-IC stimulus and extending to 500- ms poststimulus onset. Trials containing severe movement artifacts or particularly noisy events were rejected if voltages exceeded ±125 μV. Trials were then averaged to obtain grand average waveforms for 4°, 7°, and 10° IC and No-IC stimulus presentations for each subject. Median number of interpolated channels and accepted trials per condition for each group is shown in Table 2. Analyses were guided by previous IC work (44) that identified evoked response potential (ERP) effects sensitive to the difference between IC conditions during time windows associated with the visual N1.

**Table 2.** Median number of channels interpolated and number of trials accepted per group

	ASD (*n* = 32)	NT (*n* = 53)	Significance (*P*)
Channels interpolated	4 (1–11)	4 (1–11)	*P* = 0.737
Trials accepted	1375 (777–1,689)	1,419 (839–1,917)	*P* = 0.128

Median (range) are reported. Number of trials accepted includes both illusory contour (IC) and noncontour (No-IC) conditions at all stimulus sizes. ASD, autism spectrum disorder; NT, neurotypical. Statistical significance evaluated via Mann–Whitney *U* due to skewed distributions.

### Primary Analysis

#### Group comparison of IC-effect amplitudes.

Statistical analyses were implemented in SPSS (IBM Corp. Released 2020. IBM SPSS Statistics for MacOS, Version 27.0. Armonk, NY: IBM Corp.). To examine contour integration for Kanizsa figures of varying support ratios in ASD and NT participants, while limiting type-II errors, the initial analysis was restricted both spatially and temporally. We focused on electrodes over lateral occipital scalp sites (PO3 and PO4) where prior literature has suggested the strongest IC-effect responses are observed (24, 33). Data were first collapsed across both sensors of interest and diagnostic groups. The time window for the IC-effect was initially broadly defined based on component latency windows described in a previous study mapping the spatiotemporal dynamics of IC processing in NT children (44) and then further refined within these general component time windows by the grand-averaged waveforms collapsed across both groups, inclusive of IC and No-IC stimuli (i.e., without regard for or bias from the dependent measures of interest). Mean amplitudes were then computed for each subject over a 10-ms time window (184 to 194 ms) for the IC-effect. Finally, we implemented a mixed model analysis of variance (ANOVA) with a between-subjects factor of group (NT, ASD) and within-subject factors of stimulus size (4°, 7°, 10°) and hemisphere (left PO3, right PO4) to compare the IC-effect mean amplitude between groups.

### Secondary Analyses

Given that hemispheric lateralization and stimulus size did not appear to differentially modulate the IC-effect between groups, data were collapsed across all conditions for these exploratory secondary analyses.

#### Group comparison of IC-effect onset latencies.

**Figure 3.**
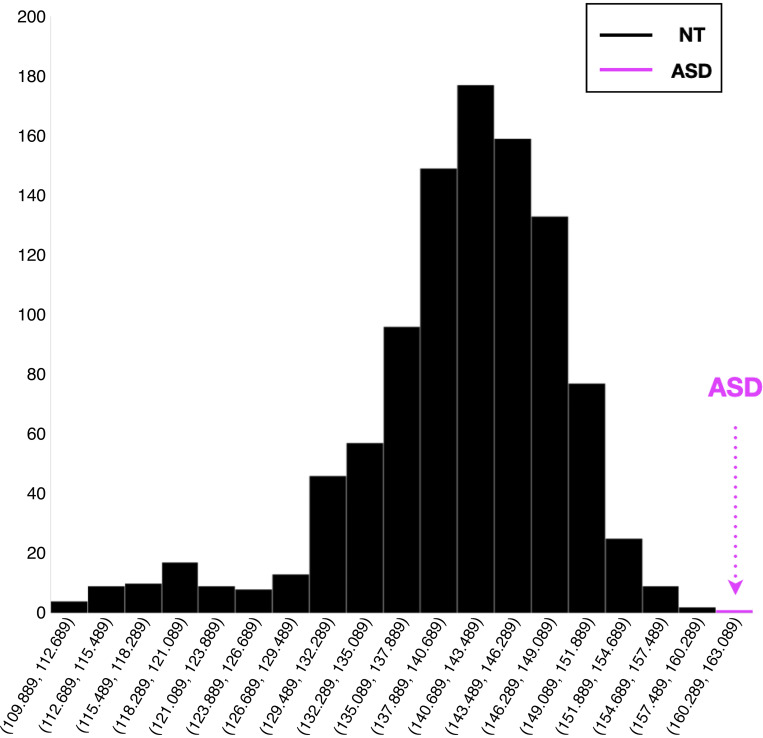
Results of the bootstrapping approach to create downsampled neurotypical (NT) cohorts with subject numbers equivalent to the autism spectrum disorder (ASD) sample. Subsets of *n* = 32 subjects were randomly selected from the NT sample iteratively to produce 1,000 combinations. The group average latency for each of these smaller NT samples was then computed using the same jackknife approach described earlier. The distribution of NT group average latency over the 1,000 combinations alongside the ASD group average latency is depicted. The observed group average latency for the ASD group (depicted in pink for reference) falls outside the full distribution of NT values.

We noted when viewing the data that although both groups generated an IC-effect that was robust in amplitude (contrary to our initial prediction), the timing of this processing appeared to differ between groups. As a result, we conducted an exploratory analysis to assess the magnitude of these latency differences. Because difference waves necessarily have a lower signal-to-noise ratio, we used a jackknife-based method to estimate onset latencies of the IC-effect. This method has been shown to outperform methods based on the selection of onset latencies at the single-participant level (48, 49). It proceeds as follows: for all *n* subjects in a given group, one subject is omitted, and the average computed over the remaining *n − 1* subjects. *n* averages are computed, each subtracting one subject’s data. For each of these *n* jackknife waveforms, an onset latency was computed. Onset latency was defined as the point between the predefined window of 50–250 ms at which the voltage reached 50% of the minimum voltage, a well-accepted estimate of onset previously used for difference-wave measures (50). Since the jackknife waveforms are digitally sampled, and thus discrete, we rarely obtained a sampled latency value that corresponded precisely with the 50% criterion. As such, we linearly interpolated between the nearest two latency values (above and below) the precise 50% voltage value. All jackknife latency measurements were conducted using ERPLAB built-in functions (47). Ulrich and Miller (49) rigorously demonstrated that the jackknife technique artificially reduces the error variances in the ANOVA. Therefore, prior to conducting the statistical analysis we used the method outlined by Smulders (51) to extract individual latencies from the jackknife average waveforms, allowing for application of traditional parametric statistical testing. These extracted latencies were then compared between groups using a Student’s *t* test. Appreciating the unequal sample size between groups, an additional follow-up analysis used a bootstrapping approach, whereby subsets of *n* = 32 subjects were randomly selected from the NT sample iteratively to produced 1,000 combinations. The group average latency for each of these smaller NT samples was then computed using the same jackknife approach described earlier. The distribution of NT group average latency over the 1,000 combinations alongside the ASD group average latency is depicted in Fig. 3 and confirms that the findings are robust to the sample size discrepancy.

#### Group comparison of VEP onset latencies.

To confirm that any changes in feedback-associated processes during the N1 latency were not due to differences at the onset of cortical visual processing, we compared the onset latency of the P1 for each group at each of the predefined electrode sites (left PO3, right PO4). For each jackknife waveform, we calculated the average onset latency for the P1 evoked by IC/No-IC stimuli. P1 onset latency was defined as the point between the predefined window of 0–180 ms at which the voltage reached 50% of the maximum voltage. As aforementioned, when a sampled latency value did not correspond with the 50% criterion, we linearly interpolated between the nearest two latency values (above and below) the precise 50% voltage value. Following the same methodology as earlier, we extracted individual latencies from the jackknife average waveforms and compared these between groups using a Student’s *t* test.

#### Sex modulation of IC-effect.

We also noted a significant difference in sex distribution between the two groups with a male predominance in the ASD group (see Table 1). Therefore, to evaluate whether sex influences the amplitude or latency of the IC-effect in children, Student’s *t* tests were performed to compare the amplitude and onset latency (calculated via the methodologies outline earlier) of the IC-effect between males and females within the NT group only.

#### Exploratory statistical cluster plots.

As the original analyses were restricted in space and time in accordance with a priori hypotheses, a secondary exploratory analysis was also performed to fully explore the richness of these high-density electrophysiological data. We computed statistical cluster plots using MATLAB to test the entire data matrix for putative effects, a method that has been effectively used by our group and others in post hoc analyses as a means to generate pointed follow-up hypotheses (52, 53). Pointwise, one-way ANCOVAs were calculated to examine differences in IC-effect between the ASD and NT group, controlling for age as a continuous covariate. *F* values for the main effect of diagnostic group were then plotted as intensity values for each time point and channel (organized by topographic region) to facilitate identification of group differences in the onset and topographic distribution of activation. To account for the increased likelihood of type I error inherent in conducting multiple comparisons, only timepoints for which the ANCOVA main effect exceeded the 0.05 *P* value criterion for at least eight consecutive data points (15.6 ms) were considered significant.

## RESULTS

### IC-effect Amplitude

**Figure 4.**
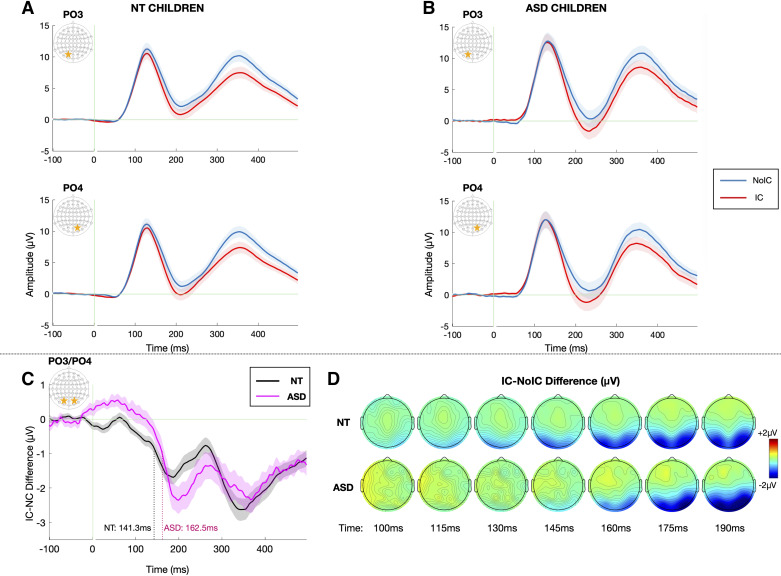
Grand average visual potentials evoked by noncontour/No-IC (blue) and illusory contour/IC (red) stimuli over the left (electrode PO3) and right (electrode PO4) lateral occipital regions for the neurotypical (NT, *A*), autism spectrum disorder (ASD, *B*) groups. Data are averaged across all stimulus sizes. Shaded regions depict standard error of the mean (SE). Green vertical line marks stimulus onset at *t* = 0. *C*: IC-effect difference wave (IC minus No-IC) averaged across PO3 and PO4. IC-effect onset derived from jackknifed measures for each group (50% of the minimum difference) is marked by dotted lines for the ASD (pink) and NT (black) groups. *D*: topographic maps depicting the IC-effect, or the difference in amplitude evoked by IC – No-IC stimuli between 100 and 190 ms for the NT (*top*) and ASD (*bottom*) groups.

**Figure 5.**
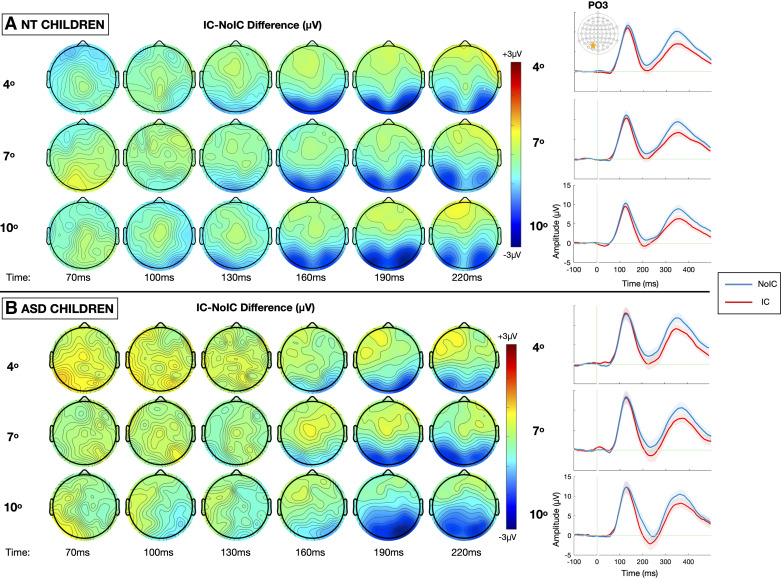
Topographic maps depicting the illusory contour (IC)-effect, or the difference in amplitude evoked by IC – noncontour (No-IC) stimuli, between 70 and 220 ms for each of the three stimulus sizes (4°, 7°, and 10° visual angle), alongside the corresponding grand average visual potentials evoked by noncontour/No-IC (blue) and illusory contour/IC (red) stimuli over the left lateral occipital region (electrode PO3) for the neurotypical (NT, *A*) and autism spectrum disorder (ASD, *B*) groups.

**Figure 6.**
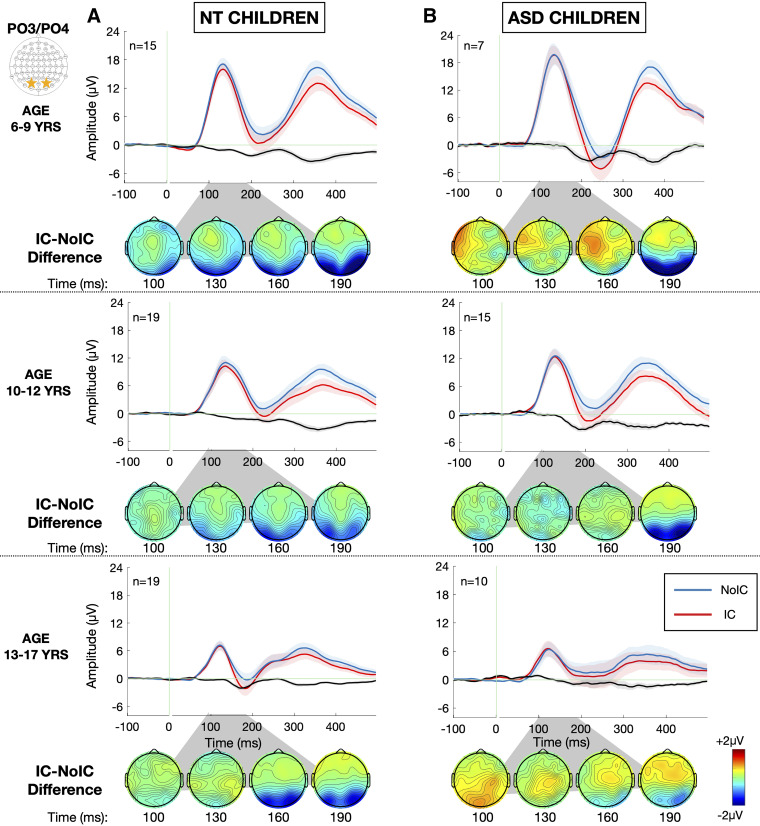
Grand average visual potentials (visual-evoked potential, VEP) evoked by noncontour/No-IC (blue) and illusory contour/IC (red) stimuli stratified by age group for the neurotypical (NT, *A*) and autism spectrum disorder (ASD, *B*) groups. VEP data are averaged over the left (electrode PO3) and right (electrode PO4) lateral occipital regions and averaged across all stimulus sizes. Shaded regions depict standard error of the mean. Green vertical line marks stimulus onset at *t* = 0. Corresponding topographic maps depicting the illusory contour (IC)-effect, or the difference in amplitude evoked by IC – No-IC stimuli between 100 and 190 ms are presented below the VEP waveforms for each age and group.

Grand-average VEPs to the IC and No-IC stimulus configurations at the a priori-defined electrodes of interest (PO3 and PO4), averaged across all stimulus sizes, are depicted for each group in Fig. 4, *A* and *B* [or see Fig. 8 for the same representation of the data excluding those participants with a DSM-4 diagnosis of pervasive developmental disorder, not otherwise specified (PDD-NOS)], and the results of the primary analysis are summarized in Table 3. As evident in Fig. 4, the IC stimuli evoked stronger negative responses than No-IC stimuli in the N1 time window in both groups, the so-called IC-effect. For the IC-effect time windows, there were no overall differences in the magnitude of the IC effect between ASD and NT groups [*F*(1,82) = 3.038, *P* = 0.089, $\eta_{\text{p}}^{2}$ = 0.034] and no group-related interactions. There was no significant hemispheric lateralization of the IC-effect [*F*(1,83) = 2.564, *P* = 0.113, $\eta_{\text{p}}^{2}$ = 0.030]. However, the magnitude of the IC-effect was modulated by size with larger stimulus size generating a stronger IC-effect [*F*(2,166) = 4.084, *P* = 0.019, $\eta_{\text{p}}^{2}$ = 0.047] in both groups. To allow for visualization of the impact of stimulus size, topographic maps depicting the differences in evoked response to IC minus No-IC stimuli for each of the three stimulus sizes (4°, 7°, and 10°), along with associated grand-average VEPs to the IC and No-IC stimulus configurations at the a priori-defined electrodes of interest (PO3 and PO4), are presented in Fig. 5. Finally, given the wide age range of the sample, the data are presented stratified by group and age in Fig. 6.

**Table 3.** Primary analysis: IC-effect amplitude

	*F*	*P* Value
*Main effects*		
Group	2.960	0.089
Hemisphere	2.564	0.113
Stimulus size (4°, 7°, 10°)	4.084	0.019*
*Within-subjects interactions*		
Hemisphere × stimulus size	1.275	0.282
*Within-subject × between-subject interactions*		
Hemisphere × group	0.500	0.481
Stimulus size × group	1.260	0.286
Hemisphere × stimulus size × Group	0.577	0.574

ASD, autism spectrum disorder; IC-effect, illusory contour effect; NT, neurotypical.

* *P* < 0.05; *n*_NT_ = 53, *n*_ASD_ = 32.

### IC-effect Onset Latency

To highlight the timing of contour integration, the difference in evoked response to IC minus No-IC stimuli at the two electrodes of interest (PO3 and PO4) for the ASD and NT groups are depicted in Fig. 4*C*, alongside topographic maps allowing for visualization of effects across the entire array (Fig. 4*D*). Onset latency of the IC-effect was estimated at 162.5 ± 27.1 ms for participants with ASD and 141.3 ± 54.5 ms for NT participants. This 21-ms delay yielded a main effect of diagnosis (*t*_83_ = 2.383*, *P* = 0.020, Cohen’s *d* = 0.458, *equal variances not assumed due to significant Levene’s Test for equality of variances) (Fig. 4*C*). In contrast to the latency of the IC-effect, onset latency of the P1 was estimated at 94.3 ± 14.3 ms for participants with ASD and 96.3 ± 32.4 ms for NT participants, and thus clearly not significantly different between groups (*t*_83_ = −3.86*, *P* = 0.747, Cohen’s *d* = −0.086, *equal variances not assumed due to significant Levene’s Test for equality of variances). Thus, NT participants’ IC processing was feedforward-dominated for ∼45 ms, whereas participants’ with ASD was feedforward-dominated for ∼68 ms.

### IC-Effect Modulation by Sex

When comparing the IC-effect between males and females within the NT group, there was no effect of sex on amplitude (*t*_50_ = −0.498, *P* = 0.621, Cohen’s *d* = −0.138) or latency (*t*_50_ = −0.686, *P* = 0.496, Cohen’s *d* = −0.190).

### Exploratory Statistical Cluster Plots

**Figure 7.**
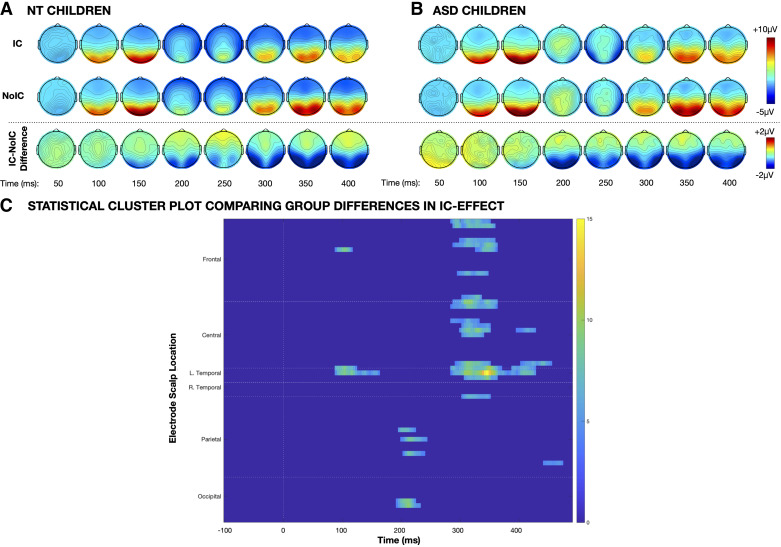
Topographic maps depicting electrophysiological activation evoked by illusory contour (IC) stimuli (*top row*), noncontour (No-IC) stimuli (*middle row*), and the IC-effect, or the difference in amplitude evoked by IC – No-IC stimuli (*bottom row*), between 50 and 400 ms collapsed across stimulus sizes for the neurotypical (NT, *A*) and autism spectrum disorder (ASD, *B*) groups. *C*: statistical cluster plot displaying regions showing statistically significant group differences in the IC-effect from a two-way ANCOVA controlling for participant age. Data are averaged across all stimulus sizes. Colors indicate the magnitude of the *F* statistic.

Dynamics of illusory contour processing across the whole scalp are depicted by topographic maps of the IC-effect in Fig. 4*D* and Fig. 5. To evaluate these dynamics, we implemented an exploratory statistical cluster analysis examining group differences in the IC-effect (averaged across all stimulus sizes) for all channels and time points with age as a continuous covariate. Results of this analysis are summarized in Fig. 7. Compared with the ASD group, the NT group displayed significantly more robust contour integration (as indexed by VEP amplitude suppression in response to IC stimuli relative to No-IC stimuli) over frontal and left temporal scalp regions during both the P1 and P3 time periods. In contrast, the ASD group displayed more robust contour integration over parietal and occipital scalp around between ∼200 ms and 250 ms.

## DISCUSSION

To investigate feedback contributions to visual processing in ASD, we compared a well-tested metric of automatic contour completion in 6- to 17-yr olds with ASD and their neurotypical counterparts. The IC-effect generated was equivalent in amplitude between ASD and NT groups. However, the IC-effect onset was 21 ms later in individuals with ASD despite simultaneous onset of visual cortical activity across the two groups. This pattern of results is suggestive of delayed visual feedback processing in ASD.

Feedforward and feedback connections between human visual cortical areas V1 and V2 seem to develop from segregated populations of neurons and follow different developmental growth patterns (18). Although feedforward axons grow toward their target cortical layers precisely, reaching them by ∼4 mo of age, feedback fibers grow past their targets, sending out multiple buds from the axon and have still not reached their targets by this time. Synaptic proliferation (54) and growth of dendritic spines (55) increase in humans over the first 5 mo of life, suggesting that space for feedback inputs may not be available until later in infancy (18, 56). Brain overgrowth prior to 3 yr of age is a consistently replicated finding in a substantial subset of children with ASD (57, 58). It has been connected to increased neuron count (59) and density (60). One possibility is that initial overexuberant feedforward connections cause feedback fibers to encounter greater obstacles to reaching their intended targets. Protracted development of feedback circuitry would likely alter the global processing or predictive functions of feedback circuitry in sensory processing (11), particularly if it is dependent on exposure gained through the earlier maturing feedforward circuitry (61).

Typically, ambiguous sensory inputs are shaped by statistical likelihoods regarding stimulus configurations acquired through prior exposure (62). Therefore, one possible implication of delayed onset of feedback-dominated activity in ASD is that perceptual representations may remain less shaped by such internal input. In this case, local features would influence initial perceptual processing for a longer time—in this case for ∼15% longer—indicating reduced influence of priors on processing of local stimulus elements. Notably, similar patterns of delayed latency but normal amplitude visual-evoked potentials have been implicated in global processing for subjects with schizophrenia, a neurological condition with shared genetic susceptibility and symptom overlap with ASD (20). It is important to note that integration of prior experience to inform visual processing can occur automatically without requirement for explicit attention (63). Indeed, visual feedback begins to influence visual processing within 50 ms prior to the time when a stimulus enters conscious awareness (19, 64). There is strong evidence that the IC-effect is one such visual process that is highly feedback-dependent, and that while contour integration representations can be detected in V1, these representations are no longer observable when visual feedback is disrupted by TMS (39). Although it is clear from multiple converging lines of evidence that contour integration depends on feedback mechanisms, definitive conclusions about the cognitive functions served by this feedback cannot be drawn. Proposed implications have included enhanced perceptual processing of detailed features (2), weakness in global processing (3), or weakened application of prior knowledge to the processing of incoming sensory data (4).

Reduced or delayed feedback does not imply only negative outcomes—that would depend on the perceptual task to which it contributes. The ambiguity of incoming features may simply be resolved later; alternatively, visual processing may adapt to more feedforward-weighted input. Such input may explain why individuals with ASD excel in tasks like the copying of geometrically impossible figures (65), where delayed feedback may mean that copying is less influenced by prior visual exposure to particular object configurations and features. Assuming this pattern predominated over childhood, this could foster development of experience-dependent visual processes that rely less on feedback mechanisms overall. In such a system it may be adaptive to place greater reliance on sensory details than on information about wholes—a characteristic of the ASD phenotype; however, additional work is clearly indicated to understand how the maturational trajectory of the visual feedback mechanisms relates to specific phenotypic characteristics in ASD. Models of predictive processing posit that in situations where sensory input is ambiguous, multiple interpretations may be actively represented in lower level cortex until feedback suppresses those determined to be less likely based on prior sensory experience (62, 66). It is possible to extrapolate that if feedback mechanisms are delayed or weakened, the sensory processing systems of individuals with ASD may be overloaded with an abundance of potential representations.

Interestingly, this work differs from prior work conducted by our group involving children in the same age range, which found a decreased amplitude of the IC-effect among children with autism, without clear latency differences (67). Although both findings point to altered feedback processing during contour integration in ASD, the different manifestations may be due to one of two categories of factors-subject heterogeneity or paradigmatic differences. Examining these areas of agreement and discrepancy are of great interest to help enrich the understanding of the factors influencing contour integration in ASD and the replicability of these findings across studies. Exploration of this type of heterogeneity represents an area of increasing emphasis for electrophysiological research in ASD (68). When comparing these two highly similar studies (67) on a number of subject factors that might influence the results, including age, sex, and IQ, the subject populations did not appear to differ on any of these factors. Consistent across both studies, along with much prior visual perception work, VEP component amplitudes were greater for younger participants in both groups. The overall larger amplitudes in younger participants may simply be attributable to anatomic features such as skull thickness (69). However, there is no evidence of age-related diagnostic group differences in the developmental trajectory of contour integration mechanisms as indexed by the IC-effect. If the IC-effect of older participants with ASD resembled that of younger NT participants, that would suggest delayed developmental maturation of contour integration in ASD. We do not observe such a pattern here. This may be more suggestive of deviant rather delayed development of contour integration processing, though a longitudinal investigation would be more ideally suited toward delineating the full developmental trajectory of contour integration across early childhood.

**Figure 8.**
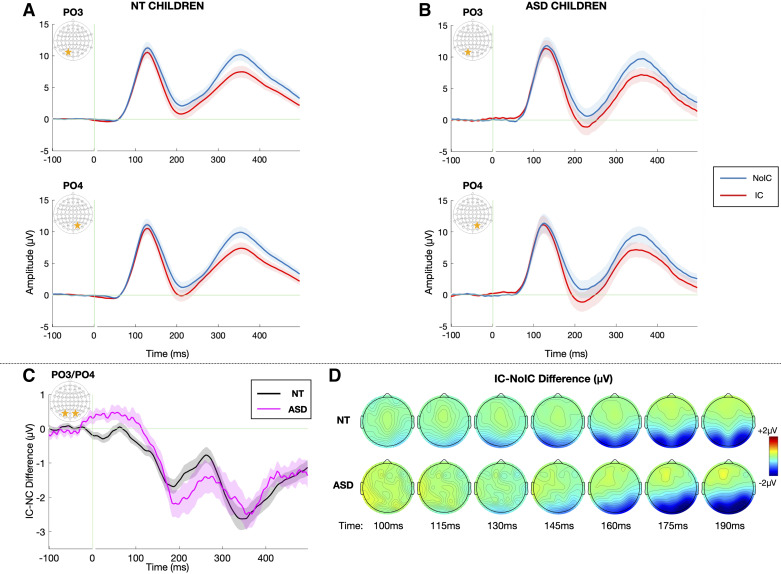
Alternate representation of the data, excluding those participants (*n* = 5) with a diagnosis of pervasive developmental disorder, not otherwise specified (PDD-NOS). Grand average visual potentials evoked by noncontour/No-IC (blue) and illusory contour/IC (red) stimuli over the left (electrode PO3) and right (electrode PO4) lateral occipital regions for the neurotypical (NT, *A*) and autism spectrum disorder (ASD, *B*) groups. Data are averaged across all stimulus sizes. Shaded regions depict standard error of the mean. Green vertical line marks stimulus onset at *t* = 0. *C*: IC-effect onset derived from jackknifed measures for each group (50% of the minimum difference) is marked by dotted lines for the ASD (pink) and NT (black) groups. *D*: topographic maps depicting the IC-effect, or the difference in amplitude evoked by IC – No-IC stimuli between 100 and 190 ms for the NT (*top*) and ASD (*bottom*) groups.

One possible phenotypic confounder is that participants in this study were diagnostically characterized prior to the implementation of the DSM-5 and are therefore classified under the older DSM-4 criteria. Although there is substantial overlap between diagnoses made under the DSM-5 and DSM-4 criteria, those diagnosed with pervasive developmental disorder, not otherwise specified (PDD-NOS) under the DSM-4 have higher rates of loss of autism diagnosis under the newer DSM-5 criteria (70). When removing this less stable diagnostic category (*n* = 5) from the results, the pattern of findings did not appear substantially different (see Fig. 8 for an alternative visualization of the data excluding those participants with a PDD-NOS diagnosis). Nevertheless, we cannot completely rule out that subject populations between this and the prior similar study (67) differed on another unmeasured factor, as autism is a highly complex and heterogeneous condition.

For example, one possibility raised in the review process is that the two samples differ in their degree of subtle trial-by-trial temporal variations in electrophysiological responses. Should temporal variations across trials be more variable either within or between individuals in the study by Knight et al. (67), the IC-effect may be more attenuated compared with that observed in the current sample. N1 attenuations due to high degrees of jitter are not unprecedented (71).

Regarding paradigmatic differences, spatial attention is potentially an interesting factor (72–74). Although both paradigms involved automatic processing of illusory contour stimuli, the prior study varied the stimulus presentation location that may have resulted in differences in covert spatial attentional orienting. Thus, the central presentations of that prior study cannot be fully isolated and compared with the present study, without the confound of spatial attention. By contrast, in the present study, spatial attention demands were limited with all stimuli being presented surrounding central fixation. Indeed, differences in spatial orienting are common in autism (75–79) and are likely an underrecognized factor in many studies of sensory perception in ASD.

Here, the main stimulus manipulation was stimulus size ranging from 4° to 10° visual angle, while holding the support ratio constant. Of note, the 4° stimulus is the same size as the IC stimuli presented by Knight et al. (67). With increasing stimulus size, we observed a stepwise increase in amplitude of the VEP components, including the N1, evoked by IC and No-IC stimuli. This is consistent with prior work suggesting increased recruitment of neurons when there is a wider central stimulus field resulting in increased VEP amplitude (80). However, the strength of the IC-effect as indexed by the difference in N1 amplitude evoked by IC versus No-IC stimuli remained constant, suggesting that the contour integration response is size invariant over this range of visual angles, again consistent with prior work (24, 81). Although one might expect larger stimuli to be biased toward involvement of higher order areas, associated with more pronounced visual feedback and augmented IC-effect, the findings are not consistent with that. However, all stimuli here did extend across the vertical meridian, requiring integration across anatomically separate visual processing regions. Our research group has previously demonstrated that presenting illusory contours laterally to central fixation, such that they no longer straddle the vertical meridian, does shift the bias toward more feedforward processing (21, 74). Most notably for the purposes of this study, the degree of size-related VEP modulation was equivalent between diagnostic groups, indicating that the ASD participants were not impaired in their ability to integrate the contours over greater visual distances.

Finally, it was raised in the review process that on post hoc examination of the data, the N1 appeared delayed across both the IC and no-IC condition in the ASD relative to NT group, despite the equivalent P1 latency between groups. The experimental paradigm was designed to examine the difference in IC minus No-IC conditions. Therefore, we did not make any a priori predictions regarding group differences in the latency of the N1 component of the VEP. Whether this finding of delayed N1 would generalize to other types of visual stimuli would be interesting to explore directly in future study. However, this does not interfere with our ability to examine the IC-effect difference waveform for group differences. The IC-effect difference waveform isolates contour integration processing from processing of the No-IC stimulus configuration, holding all features constant with the exception of illusory contour formation.

Taken together with prior literature, this electrophysiological work points to deficits in feedback-supported contour integration in ASD (67, 82) that are evident despite conflicting results behaviorally on whether IC processing deficits are present in autism. Some studies have found no difference between ASD and NT on behavioral measures of contour integration (83–85), whereas other studies have described reduced accuracy and longer reaction times in these tasks (86, 87). This discrepancy between behavioral and electrophysiological investigation suggests that individuals with ASD may accomplish equivalent IC perception, albeit via different mechanisms than their NT counterparts. Indeed, in a debriefing questionnaire, participants overwhelmingly accurately identified an illusory shape indicating that they were able to see the illusion despite the clear delays in visual feedback. Although outside the scope of the study, it would be informative to systematically compare the perceptual limit of discrimination for illusory shapes between NT and ASD groups. A recently developed paradigm using a backward masking paradigm does appear to be sensitive to detection of differences in the robustness and speed of contour integration processing and would be well suited for this purpose (38). However, it remains important to note that paradigms that rely on behavioral identification of illusory contour presence or absence necessarily force explicit attention to contour integration whereas electrophysiological paradigms allow for examination of automatic contour integration processing while attention is directed elsewhere. It remains a possibility that neural mechanisms of contour integration are augmented when participants are explicitly attending to illusory contour presence/absence, a phenomenon that has been described in other types of global visual perception in autism (88). Additional studies to compare across developmental disability populations such as ADHD and to directly assess the role of attention by comparing contour integration in attended versus passive processing would be highly interesting.

### Limitations

Although this study contributes to our understanding of the timing of contour integration processing in ASD, there remain limitations to the scope of the study that are important to highlight. For one, participants in the study represent only a portion of the extensive phenotypic variability that characterizes the autism spectrum. There is substantial overlap between ASD and ADHD. As a result, we are unable to determine whether the reduced contour integration noted in this study is specific to ASD or characteristic of ADHD or other developmental diagnoses as well. Furthermore, because participants were actively engaged in the color change discrimination task during this study, the included children needed to have the cognitive and verbal ability to understand and comply with these task instructions. Thus, results should be generalized with caution to children with so-called “profound” autism. Future adaptation of fully passive paradigms may permit the inclusion of this understudied population. Even among studies that limit samples only to participants with autism and low support needs, there are clearly areas where findings are discrepant that are extensively discussed earlier. To address this, future work should include extensive phenotypic characterization of participants to allow for better delineation of sources of sample heterogeneity. Ideally, experimental paradigms should also be designed to include a wide range of stimulus variations that all probe similar visual feedback mechanisms (e.g., inclusion of multiple variations on illusory contour, figure-ground, perceptual closure, and perceptual grouping stimuli). Directly delineating within the same participants which stimulus factors modulate group differences in neural processing would allow for a more nuanced understanding of visual feedback in ASD. In addition, mixed methodology studies such as performing psychophysical manipulations alongside EEG and/or functional imaging, and including eye tracking to verify overt attentional orienting would allow better understanding of brain-behavior linkages.

### Conclusion

Here, we demonstrate a 21-ms delay in the onset of feedback-dominated visual processing suggesting a mechanism of weakened influence of visual feedback in individuals with ASD, which may disrupt the global integration or predictive apparatuses relied on for rapid, automatic grouping of incoming sensory information.

## DATA AVAILABILITY

Data will be made available upon reasonable request.

## GRANTS

This study was supported by a grant from the U.S. National Institute of Mental Health (NIMH) under Grant No. R01MH085322 (to J.J.F. and S.M.). The Human Clinical Phenotyping Core, where the children enrolled in this study were recruited and clinically evaluated, is a facility of the Rose F. Kennedy Intellectual and Developmental Disabilities Research Center (IDDRC) which is funded through a center grant from the Eunice Kennedy Shriver National Institute of Child Health and Human Development (NICHD) under Grant No. P30 HD071593. Ongoing support of intellectual and developmental disability research, including salary support for Drs. Freedman and Foxe is provided through the University of Rochester Intellectual and Developmental Disabilities Research Center (IDDRC), funded through a center grant from the Eunice Kennedy Shriver National Institute of Child Health and Human Development (NICHD) under Grant No. P50 HD103536, and salary support for Dr. Molholm through Einstein’s RFK IDDRC, funded through a center grant from the Eunice Kennedy Shriver National Institute of Child Health and Human Development (NICHD) under Grant No. P50 HD105352. Dr. E. J. Knight is supported in part by a University of Rochester Clinical and Translational Science Institute KL2 Career Development Award KL2 TR001999 from the National Center for Advancing Translational Sciences of the National Institutes of Health. The content is solely the responsibility of the authors and does not necessarily represent the official views of the National Institutes of Health. Dr. T. S. Altschuler was supported by a Robert Gilleece Fellowship through the Program in Cognitive Neuroscience at the City College of New York.

## DISCLOSURES

No conflicts of interest, financial or otherwise, are declared by the authors.

## AUTHOR CONTRIBUTIONS

T.S.A. and J.J.F. conceived and designed research; T.S.A. performed experiments; E.J.K., T.S.A., J.W.M., and J.J.F. analyzed data; E.J.K., T.S.A., S.M., J.W.M., and J.J.F. interpreted results of experiments; E.J.K., T.S.A., J.W.M., and J.J.F. prepared figures; E.J.K., T.S.A., and J.J.F. drafted manuscript; E.J.K., T.S.A., S.M., J.W.M., E.G.F., and J.J.F. edited and revised manuscript; E.J.K., T.S.A., S.M., J.W.M., E.G.F., and J.J.F. approved final version of manuscript.
